# Third generation cognitive-behavioral therapies and genital pain

**DOI:** 10.1192/j.eurpsy.2021.1459

**Published:** 2021-08-13

**Authors:** D. Barbosa, B. Ramos, V. Covelo, M. Mota

**Affiliations:** Psychiatry, Sao Joao Hospital and University Centre, Porto, Portugal

**Keywords:** genital pain, mindfulness, acceptance and commitment therapy, compassion focused therapy

## Abstract

**Introduction:**

Genital pain is a heterogeneous chronic pain condition and the relationship between biological, psychological and social factors sets a complex clinical challenge. The importance of negative thoughts and emotions has opened up an opportunity for the role of third generation cognitive-behavioral therapies (CBT). While the majority of evidence revolves around female sexual desire and arousal problems, research on genital pain disorders is beginning to take shape.

**Objectives:**

To review the evidence of third generation CBT on genital pain disorder.

**Methods:**

Review of literature using the Pubmed platform.

**Results:**

We identified 21 publications. Evidence shows that mindfulness-based CBT (MbCBT) improves reduction of fear linked to sexual activity, pain acceptance, catastrophizing and decentering. MbCBT shows significant improvements on secondary outcomes (overall sexual function, sexual satisfaction, depression and anxiety) while reduction of genital pain has yielded contradictory results. Acceptance and commitment therapy (ACT) has been studied for chronic pain disorders with improvements on pain acceptance, psychological flexibility, anxiety, depression and functioning. Compassion-focused therapy (CFT) has yielded favorable results on pain distress and intensity, self-efficacy, self-acceptance, anxiety and depression. Self-compassion may be a promising protective factor in genital pain. Both ACT and CFT have not yet been studied specifically for genital pain.

**Conclusions:**

Third generation CBT are most commonly used for depressive, anxiety and chronic pain disorders which signals the logical role that these interventions may have in genital pain. While MbCBT has started to present favorable results in treating genital pain (as well other sexual problems), ACT and CFT require more research.

